# Sea ice drift tracks from autonomous buoys in the MOSAiC Distributed Network

**DOI:** 10.1038/s41597-023-02311-y

**Published:** 2023-06-23

**Authors:** Angela C. Bliss, Jennifer K. Hutchings, Daniel M. Watkins

**Affiliations:** 1 NASA Goddard Space Flight Center, Cryospheric Sciences Laboratory, Greenbelt, Maryland 20771 USA; 2 Oregon State University, College of Earth, Ocean, and Atmospheric Sciences, Corvallis, Oregon 97331 USA; 3 Center for Fluid Mechanics, School of Engineering, Brown University, Providence, Rhode Island 02912 USA

**Keywords:** Cryospheric science, Physical oceanography

## Abstract

A network of autonomous, ice-tethered buoys was deployed around the Multidisciplinary drifting Observatory for the Study of Arctic Climate (MOSAiC) experiment in late September 2019 for a year-long drift in the Arctic Transpolar Drift Stream. The buoys were deployed as part of the MOSAiC distributed network (DN) which included 12 multi-instrumented ice stations and an additional 116 GPS buoys distributed primarily within a 40 km radius of the MOSAiC Central Observatory. Buoy coverage within the DN was maintained with additional deployments throughout the year-long drift allowing for collection of data over a full sea ice growth and melt cycle. All GPS position data from buoys deployed within the DN have been assembled and processed into the collection of 216 quality-controlled buoy drift tracks presented in this dataset covering the period 26 September 2019 – 23 May 2021. The drift tracks in this collection are ideal for studies of dynamic sea ice motion around the MOSAiC experiment at cascading spatial scales ranging from 100s of meters to 100s of km.

## Background & Summary

The dynamic drift and redistribution of sea ice is monitored using autonomous ice-tethered buoys by tracking time series of the buoy Global Positioning System (GPS) positions^1^. Organized networks of autonomous GPS buoys deployed on sea ice are used to compute strain rates by tracking the area change from arrays of buoys over time and buoys deployed within a hierarchical network allow for analyses of the horizontal deformation (opening, closing, and shearing) of sea ice at a variety of nested spatial scales. In this data descriptor we present a data set of buoy drift tracks collected as part of the Multidisciplinary drifting Observatory for the Study of Arctic Climate (MOSAiC) experiment^2^ that are suitable for analyses of sea ice drift and deformation.

**Fig. 1:**
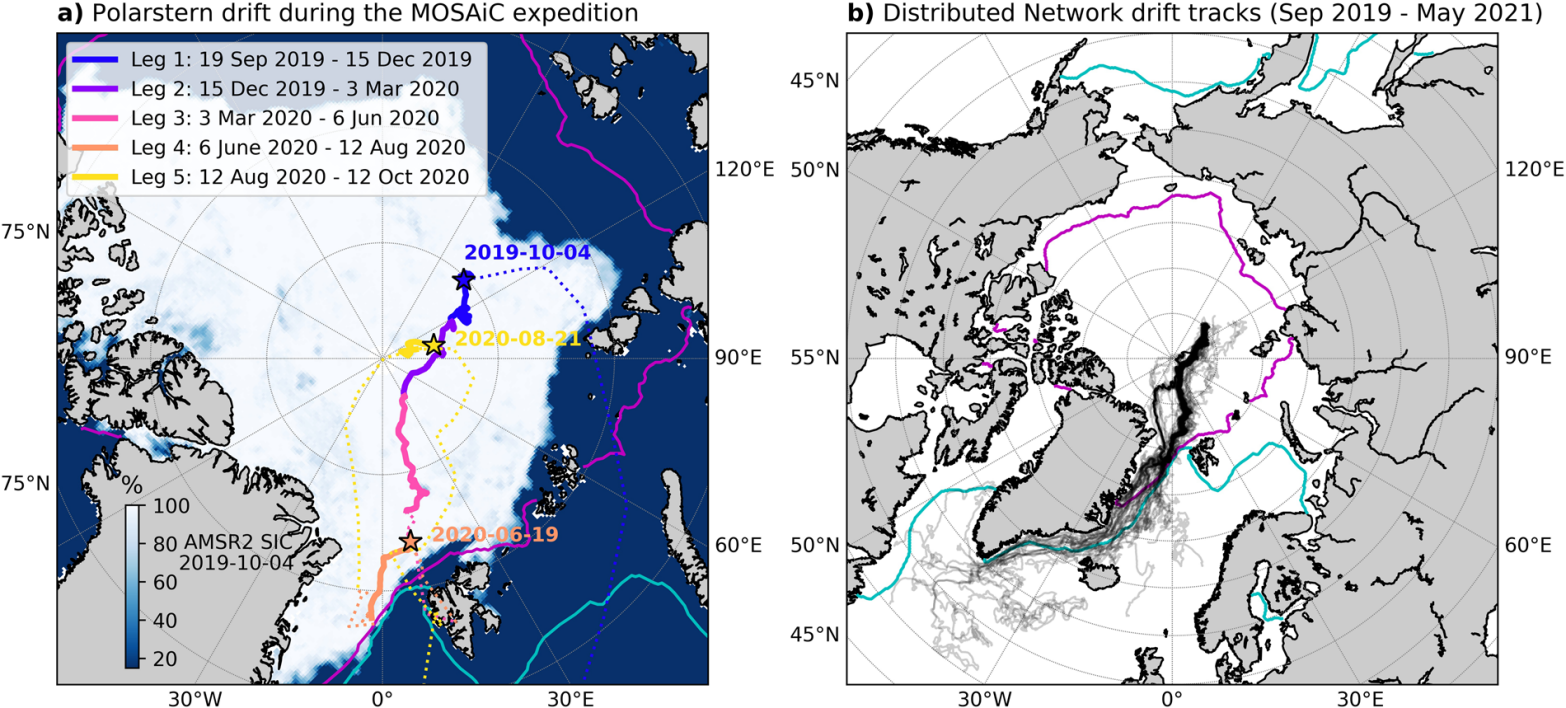
Drift of the *Polarstern* and buoys from the distributed network (DN) during the MOSAiC expedition. (**a**) The track of the *Polarstern* during legs 1–5^17–21^. Solid lines indicate passive drift of the ship, while dotted lines indicate cruise tracks. Stars indicate the starting location of drift segments. Sea ice concentration (SIC) from the Advanced Microwave Scanning Radiometer 2 (AMSR2) is shown for 4 October 2019^30^. (**b**) Full drift tracks of the 216 buoys deployed in the MOSAiC DN are shown as transparent black lines. Coloured contours show the median sea ice extent (1981–2010) in February (cyan) and September (magenta) from the National Snow and Ice Data Center Sea Ice Index, version 3^31^.

The MOSAiC field experiment was a year-long drift campaign from October 2019 – September 2020 during which the research ice breaker *Polarstern*^3^ attached to a sea ice floe in the northern Laptev Sea (85 °N, 136 °E) shortly after the onset of ice growth and began to drift across the central Arctic, eventually reaching the sea ice edge in Fram Strait (between Greenland and Svalbard) the following summer^4^ (Fig. 1a). An ice camp known as the Central Observatory (CO) was established on the floe to measure sea ice^2^, snow, ocean^5^, and atmospheric^6^ properties throughout the field experiment. The overarching goal of the MOSAiC expedition was to collect an extensive amount of field data during a year-long period to study processes in the ice-ocean-atmosphere system that impact the energy and mass balances of Arctic sea ice^2,4^; ultimately to improve representation of sea ice processes in climate models. A key component of the MOSAiC observation plan was the establishment of a distributed network (DN) of autonomous buoys which were primarily deployed throughout a 40 km radius around the *Polarstern* and the CO^2^. Buoys were deployed at greater distances around the DN in coordination with the International Arctic Buoy Programme (IABP), increasing the DN sampling area to 100s of km around the MOSAiC site, covering cascading scales of differential motion^2^. The buoys that were initially deployed in October 2019 in a 40 km radius ring around the *Polarstern* were used in the Sea Ice Drift Forecast Experiment program (SIDFEx, https://sidfex.polarprediction.net/).

This dataset contains 216 quality-controlled buoy drift tracks collected from all autonomous ice-tethered buoys deployed in the DN during the MOSAiC experiment. The drift track measurements from this data set are concentrated in the region of the Transpolar Drift Stream beginning in the high Arctic around 85 °N, north of the Laptev Sea and following the path of sea ice export through Fram Strait and along the eastern coast of Greenland (Fig. 1b). The drift data cover the period from 26 September 2019 – 23 May 2021. Each buoy drift track contains the latitude and longitude position and sampling time of the GPS instrument which can be used to determine the track and drift velocity of the buoy and the sea ice to which it is tethered. This data set was designed to be suitable for estimating strain rates and horizontal deformation from arrays of buoys, as has been done in previous sea ice field campaigns such as Ice Station *Polarstern* (ISPOL) 2004–2005 in the Weddell Sea^7^, Sea Ice Experiment: Dynamic Nature of the Arctic (SEDNA) 2007 in the Beaufort Sea^8^, and the Norwegian young sea Ice2015 (N-ICE2015) expedition in the Arctic^9^. The novelty of this collection of drift tracks from the MOSAiC experiment is that the DN was deployed and maintained for a full year, which can be used to produce a time series of strain rates for a full seasonal ice growth cycle. Further, the density of buoy deployments in the DN will allow for an analysis of sea ice drift and deformation over a range of spatial scales from 100s of m to 100s of km.

## Methods

### Buoy deployments

**Fig. 2:**
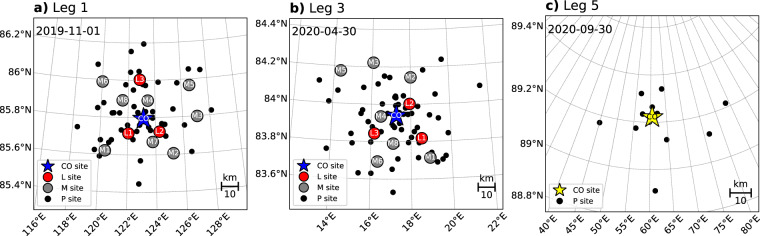
Maps of the distributed network (DN) ice station and P buoy locations. Panels show the DN layout (**a**) after initial deployment in leg 1, (**b**) after deployment of additional buoys in leg 3, and (**c**) buoys deployed in the vicinity of the Central Observatory established during leg 5 (CO3).

The distributed network (DN) of autonomous ice-tethered buoys was deployed around the drifting MOSAiC experiment in the Arctic Ocean as it traversed the Transpolar Drift Stream (Fig. 1a)^2^. A full description of the DN led by B. Rabe is in preparation; thus, only a brief description of buoy deployment sites is provided here. The DN was centered around the ice camp known as the CO which was established on the ice floe to which the *Polarstern* anchored, radiating outward from the CO at a distance of approximately 40 km (Fig. 2a). 11 remote, multi-instrumented ice stations were distributed within the DN including three large (L) sites and eight medium (M) sites (Fig. 2) and an additional distinct deployment site known as the LM site was located at a second-year ice coring site within the CO ice camp. To simplify locating the multi-instrumented CO, L, and M sites within the DN, the drift trajectories for each of the main sites were defined in a previous effort using representative GPS tracks for each site^10^. These drift trajectories of the main DN sites can be obtained from Nicolaus *et al*.^11^ In the data set presented in this paper we include all GPS tracks within each of the DN sites, allowing differential motion within these sites to be monitored. To capture horizontal ice deformation across the MOSAiC DN, GPS position buoys were distributed throughout the rest of the study area. The spacing of GPS buoys within the DN generally varies from instruments co-located to hundreds of meters apart at the L and M sites, to approximately 5 km spacing around CO and L sites and larger spacing across the full DN (Fig. 2). Additional GPS buoys were deployed at greater distances from the primary DN site (>40 km) in coordination with IABP.

The core network of DN buoys were deployed during the initial phases of leg 1 of the expedition by the *Akademik Federov* research ice breaker with helicopter support in late September and early October 2019^2^. Leg 1 deployments established the locations of CO1, L sites, and M sites within the DN (Fig. 2a). The distribution of buoys in the DN was supplemented with new deployments during leg 3 in early March 2020 to fill gaps in the network that had appeared due to instrument failure and sea ice redistribution (Fig. 2b).

*Polarstern* left the initial CO site (CO1) to complete a personnel exchange in mid-May 2020 ending the first passive drift period^2^. The ship then returned to the original CO1 floe which had since broken into smaller floes to recommence passive drift on 19 June 2020 for leg 4 (Fig. 1a). Buoys deployed in the CO following the return to the original MOSAiC ice floe are noted as deployed at CO2 (Fig. 1a). The *Polarstern* neared the ice edge in Fram Strait, and CO2 broke up on 31 July 2020 ending the second passive drift period^2^.

The ship left the sea ice for another personnel exchange, returned to the site of CO2 to retrieve buoys, then transited to higher latitudes in the Central Arctic for leg 5 (Fig. 1a). A new CO, noted as CO3, was established during leg 5 when *Polarstern* began a third drift at a new ice floe on 21 August 2020^2^. Some buoys were deployed as the ship transited to the new CO location and a smaller DN of buoys were deployed around CO3 (Fig. 2c). The third passive drift segment and the crewed portion of the MOSAiC experiment ended on 20 September 2020 when the *Polarstern* left the ice floe to return to Bremerhaven, Germany^2^. The remaining autonomous buoys from the second DN deployed during leg 5 continued to collect data through early 2021.

### Instrumentation

Several types and models of autonomous buoys and instruments were deployed in the DN to monitor sea ice drift, snow and ice thickness and temperatures, atmospheric conditions, radiation, and oceanic conditions. The instruments are grouped into 12 general categories for buoy type including: atmospheric surface flux station (ASFS), ocean conductivity, temperature, and depth buoy (CTD), drift-towing ocean profiler (DTOP), autonomous ocean flux buoy (Flux Buoy), modular sea ice buoy (IMBFlex), ice-tethered profiler (ITP), optical chain and logger (OptiCAL), radiation station, seasonal ice mass balance buoy (Seasonal IMB), snow height beacon (Snow Buoy), surface velocity program (SVP) drifters, and thermistor string buoy (Thermistor). Table 1 summarizes the specifications for buoys in these categories including the buoy type, description, manufacturer, model, and, where possible, the manufacture’s stated GPS receiver accuracy. Records in the Alfred Wegner Institute (AWI) Sensor Information System (https://sensor.awi.de/; Last accessed: 4 October 2022) used to document instrument deployments at MOSAiC inaccurately describe the OptiCAL buoys as Light Ice Tethered Profilers (LITO) or Environmental Pope (Envipope) buoys (Table 1). In this dataset we use the name OptiCAL to identify the optical chain buoys manufactured by SAMS Enterprise Ltd. Additionally, users should note that the ASFS are not buoys; rather, they are sled-mounted instrument packages that were deployed on the sea ice, operated autonomously, and were manually repositioned on occasion. However, for the purposes of this dataset, we refer to all above instruments as buoys.

**Table 1:** Specifications for autonomous, ice-tethered buoys deployed in the MOSAiC Distributed Network.

Buoy Type^a^	Description	Manufacturer	Model	GPS Receiver (accuracy)	Raw Data Source
ASFS	Atmospheric Surface Flux Station	—	—	Hemisphere GPS Compass V102 (1.2 m)	Data Provider Chris Cox^26–29^
CTD (O)	Ocean Conductivity, Temperature, and Depth Buoy	PacificGyre IT	SVP5S	uBlox Lea M8	PacificGyre IT data portal^b^
DTOP Ocean Profiler (V)	Drift Towing Ocean Profiler	Tianjin Yuanxun Technology Co. Ltd.	DTOP Ocean Profiler	—	meereisportal.de
Flux Buoy (F)	Autonomous Ocean Flux Buoy	Naval Postgraduate School	Autonomous Ocean Flux Buoy	Trimble Copernicus II	meereisportal.de
IMBFlex (M)	Modular sea ice buoy	Bruncin	IMBFlex	S1315R (±2.5 m)	meereisportal.de
ITP (W)	Ice-Tethered Profiler	Woods Hole Oceanographic Institution	Ice-Tethered Profiler	Navman Jupiter (2.1 m)	meereisportal.de
OptiCAL (E)	Optical Chain and Logger	SAMS Enterprise Ltd.	OptiCAL	Adafruit Ultimate GPS Breakout (<3 m)	meereisportal.de
Radiation Station (R)	Radiation Station	Bruncin	Radiation Station	—	meereisportal.de
Seasonal IMB (I)	Seasonal Ice Mass Balance buoy	CryoInn	SIMB3	MTK3339 (3 m)	meereisportal.de
Snow Buoy (S)	Snow height beacon	MetOcean Telematics	ICEB-I-TBAS-SH-A	Navman Jupiter 32xLP (±3 m)	MetOcean Telematics data portal^c^
SVP (P)	Surface Velocity Program	MetOcean Telematics	SVP-I-XXGS-LP	Telit Jupiter JF2 (±2.5 m)	MetOcean Telematics data portal^c^
			SVP-I-BXGS-P	Telit Jupiter JF2 (±2.5 m)	JouBeh Technologies data portal^d^
			SVP-I-BXGS-AP	Telit Jupiter JF2 (±2.5 m)	MetOcean Telematics data portal^c^
		PacificGyre IT	Ice Tracker	uBlox Lea M8	PacificGyre IT data portal^b^
			Universal Tracker	uBlox Lea M8	PacificGyre IT data portal^b^
			SVP-B	uBlox Lea M8	PacificGyre IT data portal^b^
		Taiyuan University of Technology (TUT)	iSVP	—	PI Ruibo Lei, personal communication
		MarlinYUG	iceST/30	GlobalTop Titan3 (±3 m)	meereisportal.de
Thermistor (T)	Thermistor string buoy	SAMS Enterprise Ltd.	SIMBA V7	Fastrax UP501 (±2 m)	SAMS Enterprise Ltd. data portal^e^

^a^The letter in parenthesis is the corresponding indicator of buoy type used in the Sensor ID code (see text).

^b^https://api.pacificgyre.com/api2/,

^c^ftp://ftp.metocean.com/,

^d^ftp://ftp.joubeh.com/,

^e^https://simba.srsl.com/.

During MOSAiC, the CO1, CO2, CO3, and LM sites were equipped with a variety of buoy types including flux buoys, Seasonal IMB, OptiCAL, IMBFlex, radiation station, snow buoys, thermistor, CTD, an ASFS sled and SVP buoys (See CO_site_buoy_summary.csv deposited at Arctic Data Center^12^). The three L sites were instrumented with flux buoys, Seasonal IMB, radiation station, snow buoys, thermistor, ITP, and ASFS sleds (See L_site_buoy_summary.csv deposited at Arctic Data Center^12^). The eight M sites were equipped with CTD profilers, snow buoys, thermistor and DTOP ocean profiler buoys (See M_site_buoy_summary.csv deposited at Arctic Data Center^12^). The remainder of the DN included the P site buoys that were distributed throughout the study area. The 116 P site deployments in the DN included 110 SVP buoys, six thermistor, and 2 snow buoys (See P_site_buoy_summary.csv deposited at Arctic Data Center^12^).

The buoys deployed in the DN collected a variety of measurements during the MOSAiC experiment; however, to construct the database of buoy drift tracks, we use only the GPS position data.

### Drift track processing and quality control

**Fig. 3:**
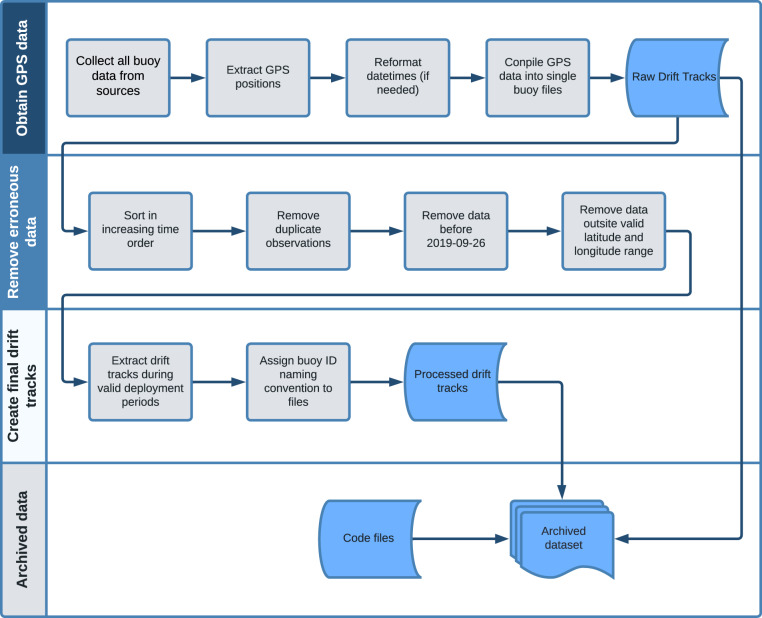
Flowchart of distributed network drift track processing. Blue shapes indicate steps in the processing that produce files archived in the dataset including the raw GPS data, the final processed drift tracks, and the code used to produce the dataset.

Processing of the buoy drift track dataset involved collecting the raw GPS position data from all DN buoys, minor cleaning of the drift data, and assigning track identification metadata to the data files. A flowchart of the drift track data processing is shown in Fig. 3 and the methodology is described in detail below.

#### Obtain GPS data

A significant portion of the development of this dataset involved obtaining the raw data for each buoy from one of three source types. The ASFS data and data from SVP buoys manufactured by Taiyuan University of Technology (TUT) were obtained directly from the data providers or instrument principal investigators (PIs) for use in this data set. The CTD, Snow Buoy, Thermistor, and SVP models manufactured by MetOcean Telematics, PacificGyre IT, and SAMS Enterprise Ltd were downloaded directly from the manufacturer’s online data portals (Table 1) with private login credentials. Raw data files for all other buoys were downloaded from the www.meereisportal.de MOSAiC buoy data portal (https://data.seaiceportal.de/gallery/index_new.php?lang=en_US&active-tab1=mosaic&active-tab2=buoymosaic; Last accessed: 22 August 2022) where preliminary buoy data from MOSAiC is hosted. The raw data source for each buoy type/model is provided in Table 1.

The raw data included all measurements made by the instrument. We extract the GPS position observations including the latitude, longitude, and observation datetime from the files, reformat the datetimes if needed to a consistent format (YYYY-MM-DD HH:MM:SS) in UTC time, and compile the data into a single file per buoy (Fig. 3). The resulting raw drift track files are saved and archived at the Arctic Data Center^13^.

#### Remove erroneous data

The procedure used to clean the raw GPS data is the same methodology used by prior studies that derived horizontal sea ice deformation from buoy networks in the Arctic^8,9^ and Antarctic^7^ with further refinement as described in the next section. As a first pass to remove obviously erroneous data the raw drift track files from step 1 were sorted and filtered in the following order (Fig. 3).

1. The observations are sorted in increasing time order.
2. Duplicate observations are flagged and removed.
3. Any observations prior to the first date of MOSAiC buoy deployments (26 September 2019) are removed.
4. Remove any observations with a latitude or longitude value outside of the valid range. Valid latitudes and longitudes range from 50 °N to 90 °N and −180 °W to 180 °E, respectively.

It should be noted that removing observations that fall outside of the fixed valid latitude range confines drift tracks to the Arctic domain but does not confine tracks to the sea ice region. The sea ice edge is well north of 50 °N latitude; thus, GPS observations were collected from buoys that were no longer tethered to sea ice and were instead drifting in the ocean as can be seen in Fig. 1b. To support broad reuse of the data set in other applications, we do not apply further filtering to restrict the observations to only sea ice regions. However, users should be aware that the ends of some drift tracks are not representative of sea ice drift.

#### Create final drift tracks

Many of the buoys in the MOSAiC DN were turned on and collecting data before their deployment on the ice and several buoys were recovered and then redeployed at a new location. Thus, we extracted the data during valid buoy deployment periods from the cleaned time series. The timeseries for one buoy deployment begins with the first timestep following the deployment time and either [1] the last timestep when a good buoy position was reported before instrument failure or [2] the last timestep before the buoy was physically recovered. Buoy deployment and recovery times were obtained from entries in the buoy action log registered in the Alfred Wegner Institute (AWI) Sensor Information System (https://sensor.awi.de/; Last accessed: 4 October 2022) during the field experiment or from deployment reports archived at www.meereisportal.de (Last accessed: 22 August 2022).

Once the valid data during deployment periods are extracted, the final processed buoy drift tracks are saved to individual files with a descriptive file naming convention to identify the deployment site, the buoy, and a unique deployment code. The processed drift tracks are archived at the Arctic Data Center^12^.

Additional summaries of each buoy drift track in the dataset are found in four summary tables organized by deployment sites (i.e., CO, L, M, and P sites) deposited with the drift track data set at Arctic Data Center^12^. These tables provide the buoy ID numbers, buoy type and primary sampling frequency; deployment date and location, track duration (d), distance travelled (km), and mean drift speed (m/s) computed with a backward difference method. For many buoys, the regular sampling frequency varied over the deployment period; thus, the sampling frequencies reported in the summary tables, represent the highest frequency reporting interval of the drift track.

## Data Records

Processed drift tracks for individual buoys deployed in the DN and the raw GPS position files associated with each buoy are provided in comma-separated value (.csv) format. The processed data files contain sorted records of the date and time of the position observation in UTC, latitude, and longitude. Additional metadata providing details on the buoy type, manufacturer, model, sampling frequency, buoy owner or project PI, and authors of the source data for each drift track are provided in a separate .csv file that is archived with the processed drift tracks (DN_buoy_list_v2 .csv).

The full record of processed drift tracks from autonomous buoys in the MOSAiC DN from 26 September 2019 through 23 May 2021 is archived in the Arctic Data Center data repository^12^. Additionally, all archived data associated with this MOSAiC DN buoy drift track data set including the raw buoy data and processing code can be accessed at the Arctic Data Center Data Portal website (https://arcticdata.io/catalog/portals/MOSAiCDriftTrack).

The .csv files for the processed data use descriptive filenames to identify the drift track data contained in the file. Filenames in the data set follow the structure “DNID_IMEI_SensorID.csv” where the DNID, International Mobile Equipment Identity (IMEI), and SensorID components correspond to the deployment site, instrument, and unique chronological deployment code, respectively, of the specific drift track contained in the file. The codes used in the file naming convention are described further in Table 2 and the following section.

**Table 2:** Summary of the MOSAiC DN drift track data set file naming convention and associated identification codes.

Filename Variable and Identification Code	Description	Purpose	Format or Pattern	Unique to a single buoy track?^a^
DNID	Distributed Network Identification code	Indicates the ice station or deployment position within the DN (see Fig. 2)	CO configurations (Fig. 1): CO1 – CO3; L sites: L1 – L3; M sites: M1 – M8	No
			P sites throughout the DN: P001 – P120	Yes
IMEI	International Mobile Equipment Identity^b^	Indicates the unique buoy or instrument hardware number	15-digit numeric code	No
			For ASFS stations: ASFS30UCB, ASFS40UCB, ASFS50UCB, or ASFSTUCB	No
Sensor ID^c^	AWI Sensor Information System Identification code^b^	Identifies the deployment event for each buoy track	YYYYBNNN, where YYYY is the deployment year, B is the buoy type code (see Table 1), and NNN is a chronological number of the event	Yes
			For ASFS stations: ASFSXXUCBN, where XX is the ASFS station as above and N is a number indicating repeated deployments in chronological order.	Yes
.csv	comma separated value	File type	—	—

^a^”Yes” if the ID code is unique to a single buoy drift track file in the data set. “No” if the ID code applies to one or more tracks in the data set.

^b^Except for ASFS stations which use an alternative code. See text for more details.

^c^The Sensor ID code is always unique to a specific drift track. This code is the best identifier for a specific drift track in the data set.

### Buoy and drift track identification codes

Specific buoy drift tracks or all tracks associated with a unique buoy can be identified in the data set using the identification codes in the filenames of the processed .csv files. The three identification codes used in the data set are described in detail below to help users identify specific tracks or buoys of interest and are summarized in Table 2.

#### DN ID code

The first of the three identification codes, the DN ID, indicates the ice station or position of deployment within the DN (i.e., Fig. 2). DN ID codes for ice stations include: L1, L2, and L3 for the three large “L sites”; M1 through M8 for the eight medium “M sites”; LM for a distinct second-year ice coring site within the main CO used during the MOSAiC field campaign; and CO1, CO2, and CO3 for the three Central Observatory configurations (Fig. 1a) installed during MOSAiC. As described above, the DN ID CO1 identifies buoys deployed during legs 1–3 within the CO established at the first MOSAiC ice floe. *Polarstern* left the CO1 site and first ice floe to exchange personnel between legs 3 and 4. CO2 identifies buoys that were deployed after the *Polarstern* returned to the initial CO1 site during leg 4. CO3 identifies buoys deployed at a new ice floe in the CO that was established when the *Polarstern* repositioned for leg 5 following breakup of the initial MOSAiC floe at the sea ice edge in late July 2020.

GPS buoys that were distributed throughout the DN, in contrast to groups of buoys deployed at ice stations (e.g., L and M sites), are known as “P buoys” for point or position buoys. The DN ID code for P buoys are numbered and preceded by a “P”. P buoys numbered P001 – P069 were deployed in leg 1, P070 – P087 were deployed in leg 3, and P090 – P120 were deployed in leg 5.

#### IMEI number

The IMEI number is a 15-digit identification number that is unique to a specific buoy or instrument. During MOSAiC, many of the buoys were recovered and redeployed elsewhere in the DN at a later time; thus, the IMEI, like the DN id codes, cannot be used as a unique identifier for drift tracks. In this dataset the IMEI number identifies the instrument that collected the data for the drift track.

#### Sensor ID code

The Sensor ID code is an identification code used to identify deployments of instruments on the AWI Sensor Information System (sensor.awi.de). All actions related to buoy deployment, servicing, recovery, and failure during the MOSAiC field campaign were registered in a tracking system and linked to the buoy Sensor ID code assigned on deployment.

The Sensor ID code is composed of the deployment year (2019 or 2020), a single letter code to describe the general buoy type, and a number that corresponds to chronological deployment order collective of all instruments deployed during the MOSAiC expedition. Buoy types are codified in the Sensor ID with the letter listed in the buoy type column of Table 1 (e.g., “S” for Snow Buoy, “P” for SVP Buoy, or “T” for Thermistor Buoy). The Sensor ID code is unique to each buoy deployment; therefore, it is the only ID code that can be used to identify a specific drift track aside from the numbered DN ID codes for P buoys.

All buoy drift tracks in this dataset are associated with a Sensor ID code that was assigned on deployment with the exception of tracks from the ASFS. The ASFS are not buoys, but rather instrumented sleds that were deployed to collect a suite of atmospheric and radiation data at each of the L sites and in CO2 and CO3. The ASFS collected high frequency GPS positions which provide highly accurate drift tracks in this data set. However, the ASFS stations were not assigned Sensor ID codes like other buoys in the DN and do not have an associated IMEI number. Instead, we identify drift tracks from ASFS deployments using alternate codes unique to this data set. The three ASFS stations and one flux tower are identified as ASFS30UCB, ASFS40UCB, ASFS50UCB, and ASFSTUCB (“T” for tower) used in place of an IMEI number and appended with a number to differentiate redeployments of the systems in chronological order. For example, “ASFS50UCB3” is the code used to identify the third deployment of the ASFS50UCB station in lieu of a Sensor ID code.

## Technical Validation

Current GPS positioning systems have accuracies of less than 3 m in the Arctic^14^ (Table 1). Buoys deployed at the 12 ice stations (COs, L and M sites) were deployed within short distances and sometimes collocated, while P buoys were deployed around ice stations with a spacing density of 5 km or more. Horizontal accuracies of ±3 m are well within the range of accuracy considered adequate for studies of sea ice drift and deformation^15^. To further validate the accuracy of the buoy drift tracks, we compare the tracks from buoys in the DN near the *Polarstern* where similar ice conditions were present.

In the early autumn when the DN was deployed in leg 1, the sea ice within a 40 km radius of CO1 was a collection of loosely assembled, first-year ice floes that had survived the summer melt season^4^. The sea ice in the region was generally heterogeneous, however, sea ice within ~40 km of the CO was found to be 36% thinner than that in an extended region around the MOSAiC site^4^ (220 km radius). As the autumn and winter seasons progressed, the ice pack in the region around the *Polarstern* thickened and consolidated through October and November 2019 and the observed dynamic ice motion decreased in December 2019 and January 2020^16^. Dynamic ice motion increased to moderate levels in the early spring and increased suddenly as the MOSAiC site approached the marginal ice zone^16^ during leg 4 (Fig. 1a). Therefore, we expect that the drift of DN buoys in the vicinity of *Polarstern* (40 km radius) should have similar drift trajectories, especially during periods where the sea ice is fairly consolidated (e.g., autumn and winter) and be less consistent when the sea ice is dynamically active and broken up such as during the summer melt season and as the DN neared the marginal ice zone during leg 4.

**Fig. 4:**
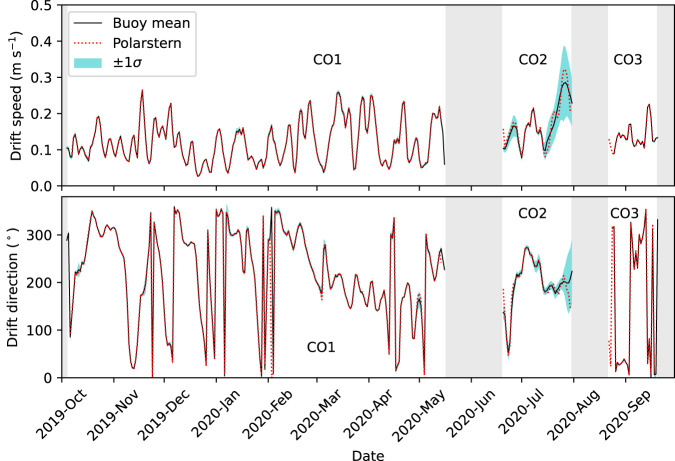
Variability in buoy drift speed and drift direction around the *Polarstern*. The variability in daily mean drift speed (top) and direction (bottom) for DN buoys in the vicinity of Central Observatory (CO) sites is compared to the daily mean drift of the *Polarstern* smoothed with a 3-day running mean. Cyan shading shows ±1 standard deviation (σ) around the mean of buoy drift. Grey shading denotes *Polarstern* transit periods.

To assess the variability in buoy drift tracks from the DN, we compare the mean drift speed and direction of buoys within the primary core of the DN to the drift of *Polarstern* during passive drift periods at CO1 (legs 1–3), CO2 (leg 4), and CO3 (leg 5; Fig. 4). DN buoys deployed in the vicinity of *Polarstern* (~40 km radius around CO1 and CO2 and ~2 km radius around CO3) and with observation frequency of 4 hours or less were selected for comparison during each drift. Daily time series of drift speed and direction were obtained for each buoy track using a backward difference method. The drift speed was computed by dividing the geodesic distance based on the WGS-84 ellipsoid between two consecutive buoy position observations by the time step in s between the observations. The computed speed is then assigned to the later time step and is expressed in ms^−1^. Drift direction was computed as a forward azimuth toward the later time step and expressed in degrees with north at 0°. The circular mean and standard deviation of the drift speed and direction of the selected buoys for each CO was then computed. The daily mean *Polarstern* drift speed was computed as above using master tracks from the *Polarstern* MOSAiC cruises at 10 minute resolution^17–21^ and the direction was obtained from the *Polarstern* course field provided in the master track data. Figure 4 compares buoy drift speeds (top) and buoy drift direction (bottom) to the *Polarstern* drift speed and direction, respectively, for the three passive drift periods of MOSAiC smoothed with a 3-day running mean. The mean of buoy drift in the vicinity of *Polarstern* during the passive drift periods at CO1, CO2, and CO3 are shown with the solid black curves. The cyan shading shows ±1 standard deviation to illustrate the variability of the buoy drift observed around the ship.

When compared to the *Polarstern* (dotted red curves), the mean buoy drift speed and direction (black curves) are very similar indicating that the surrounding ice is drifting in a similar trajectory to that of *Polarstern* during the CO1 and CO3 drift periods (Fig. 4). When higher standard deviations in drift direction are apparent (cyan shading; Fig. 4 bottom), there are slight deviations between the mean buoy and *Polarstern* drift direction. This result suggests that for some cases, shifts in the drift direction are non-uniform across the DN affecting individual buoys over a range of time. These occurrences may be related to effects of storm or wind activity resulting in deformation of the sea ice pack^22^. The largest discrepancies in speed exist for CO2 where there are slight differences between the mean buoy drift and the *Polarstern* drift (<0.5 cm/s). CO2 is also the period in which the largest standard deviations in buoy drift speed (0.1 ms^−1^) and direction (68.5°) occur. The CO2 drift occurred in leg 4 of the MOSAiC expedition (Fig. 1a) when the *Polarstern* and the DN was located nearest the sea ice edge and the marginal ice zone in the summer of 2020 when the ice was broken up and less consolidated^16^. Therefore, the increased variability of buoy drift speeds and direction (i.e., larger standard deviation) and larger differences between the mean buoy drift and *Polarstern* drift is consistent with what we expect for sea ice conditions during the summer melt season and as the DN passed through Fram Strait.

The close agreement between the *Polarstern* drift and the mean of buoys in the vicinity of the ship and COs, during the more consolidated sea ice periods (CO1 and CO3) and increased variability observed during CO2 corresponds well with the expected drift differences of more broken up floes that would be present with coincident sea ice conditions. This result gives us confidence that the buoy GPS drift tracks are accurately representative of the sea ice drift conditions in the DN. The drift comparison in Fig. 4 should guide users in understanding how cohesive the drift of ice floes around the *Polarstern* was during each leg of the drift experiment (Fig. 1a).

## Usage Notes

In most cases users should apply methodology to interpolate buoy drift tracks to the expected sampling frequency prior to conducting analyses with more than one drift track at a time. Most buoys in the DN sampled hourly, every half hour, or every 10 minutes with reports beginning at the top of the hour. SVP buoys manufactured by TUT are an exception which reported hourly GPS positions at the half hour. Slight delays in the reporting of the buoy via iridium satellite results in observation times that are a few seconds different from the official reporting time. Thus, users should apply an interpolation scheme of their choice (e.g., interpolating to hourly observations) such that buoy observations begin at the top of the hour to enable easier comparison of the positions of different buoys at the same time stamp. However, it is important to note that interpolating drift tracks from buoys that sampled less frequently (e.g., every 3, 4, or 12 hours) to hourly or finer time scales may produce unexpected results in the drift tracks.

Although the expected GPS accuracies of buoy drift tracks are 3 m or less^14^ (Table 1), random errors in GPS positions from drifting buoys do occur^23^. Users should be aware that position outliers in the drift tracks are not filtered out in the minor data cleaning procedures applied here.

## Data Availability

The Interactive Data Language (IDL) code and Python and shell scripts used to produce the processed DN buoy drift tracks are archived and available for download at the Arctic Data Center^24^.
